# Preoperative diagnosis of obscure gastrointestinal bleeding due to a GIST of the jejunum: a case report

**DOI:** 10.4076/1757-1626-2-8186

**Published:** 2009-09-10

**Authors:** Stavros Gourgiotis, Dimitrios Kotoulas, Stavros Aloizos, Aikaterini Kolovou, Nikolaos S Salemis, Ioannis Kantounakis

**Affiliations:** 1 Second Surgical Department401 General Army Hospital of AthensGreece; 2 Intensive Care Unit401 General Army Hospital of AthensGreece; 3 Radiology Department401 General Army Hospital of AthensGreece

## Abstract

Gastrointestinal stromal tumours are rare mesenchymal neoplasms affecting the digestive tract or nearby structures within the abdomen. We present a case of a 66-year-old female patient who presented with obscure anemia due to gastrointestinal bleeding and underwent exploratory laparotomy during which a large gastrointestinal stromal tumour of the small intestine was discovered. Examining the preoperative results of video capsule endoscopy, computed tomography, and angiography and comparing them with the operative findings we discuss which of these investigations plays the most important role in the detection and localization of gastrointestinal stromal tumours. A sort review of the literature is also conducted on these rare mesenchymal tumours.

## Introduction

Gastrointestinal stromal tumours (GISTs), which arise primarily in the gut wall, are uncommon mesenchymal, malignant or potentially malignant tumours affecting the gastrointestinal tract. GISTs are the most common non epithelial tumors of the digestive tract, accounting for only 1% of all gastrointestinal malignancies [1,2] and for 5.7% of all sarcomas [3]. These tumours are defined as specific, generally Kit (CD117)-positive and Kit or platelet-derived growth factor receptor alpha (PDGFRA) mutation-driven tumours [4].

This paper reports a case of obscure gastrointestinal bleeding due to a large GIST of the jejunum evaluates the role of preoperative investigations of this association and provides a short English literature review.

## Case presentation

A 66-year-old Caucasian female with no past medical history presented with a 3-month history of anemia. There was no associated fever, dyspeptic symptoms, nausea, weight loss, vomiting, or localized abdominal pain. Physical examination and blood biochemistry were within normal rates. Hematologic tests showed decrease of hematocrit (Ht: 19%) and platelets (PLT) count of 76,000/μL. The patient was HIV 1-2 negative. CEA and CA 19-9 were in the normal range.

**Figure 1.**
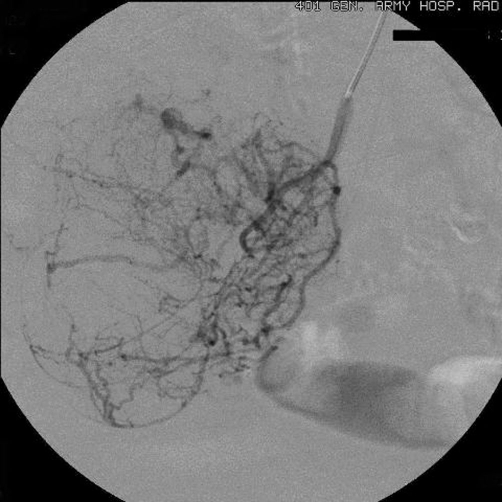
The superselective catheterization of an ileal artery shows voluminous disorder with abnormal arterial structures and areas of stagnation of the contrast medium.

**Figure 2.**
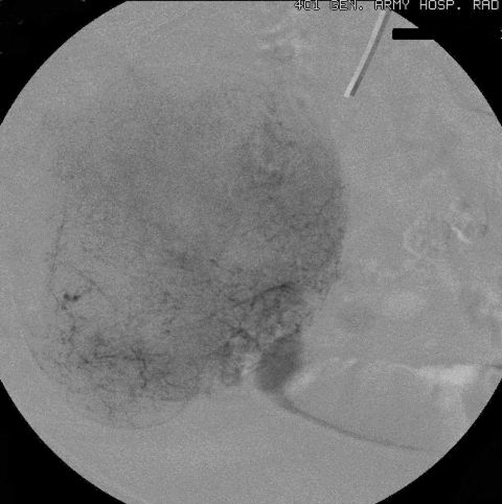
The parenchymal phase of the superselective catheterization of the same ileal artery shows the full extent of the disorder.

Chest and abdominal X-rays, abdominal ultrasonography (US), upper gastrointestinal endoscopy, and colonoscopy were unremarkable. Abdominal computed tomography (CT) showed a well-delimited lobulated large mass measuring 10 × 9 × 5 cm in the jejunum with malignant behavior. Video capsule endoscopy (VCE) revealed an extensive venous plexus giving the possible diagnosis of angiodysplasia of small intestine. Angiography demonstrated voluminous disorder with abnormal arterial structures and areas of stagnation of the contrast medium (Figures 1,2). Due to the extension of the disorder, the embolization was abandoned.

**Figure 3.**
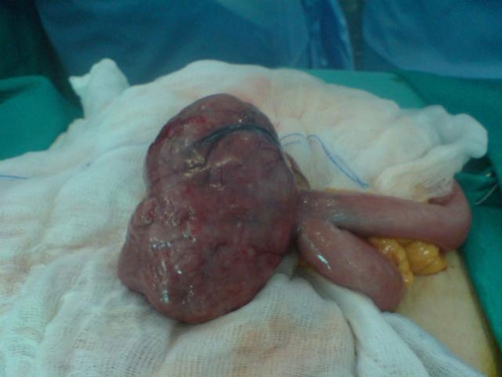
The well-circumscribed lesion of the jejunum before its removal.

The patient underwent exploratory laparotomy. During surgery, a well-circumscribed lesion, measuring 10.5 × 9.5 × 6 cm was identified at the jejunum (Figure 3). A complete resection of the mass and a side to side anastomosis of the small bowel were performed. The patient had an uneventful postoperative course and was discharged on the sixth postoperative day.

The histopathological examination of the resected lesion revealed a mesenchymal tumour categorized as GIST tumour. The stromal tumour demonstrated whirling sheets of spindle cells with moderate level of phenomorphism and mitotic activity (6-7 mitoses/50 HPF) (H&E stain). No necrosis was observed. Immunohistochemical staining for CD117, α-smooth-muscle actin (SMA), and S-100 protein was positive, whereas staining for desmin and CD34 was negative. The labeling index for MIB-1, determined by counting positively stained nuclei, was about 5%.

## Discussion

The term GIST was introduced by Mazur and Clark in 1983 in order to indicate a distinct heterogeneous group of mesenchymal neoplasms of spindle or epithelioid cells of varying differentiation [5]. GISTs account for only 1-3% of gastric tumours, 20% of small bowel tumours, and 1% or less of colorectal tumours. They have a predilection for adults older than 50 years, with the median ages varying around 60 years. Although these tumours usually develop in a sporadic fashion, familiar occurrence has also been reported [6].

Random genetic mutations are the apparent cause of GISTs. The majority of these tumours show identified mutations in cell-surface proteins called tyrosine kinase receptors [4]. Most GISTs show mutations in a gene that produces a growth factor receptor called KIT [6].

Primary GISTs may occur anywhere along the GI tract from the esophagus to the anus [7]. The most frequent site is the stomach (55%), followed by the duodenum and small intestine (30%), esophagus (5%), rectum (5%), colon (2%), and rare other locations.

The most common presentation of GIST is acute or chronic gastrointestinal bleeding. They often present with nausea, vomiting, abdominal pain, metastatic diseases, and bowel obstruction. However, the symptoms depend on GIST location and size. Many tumours are found incidentally through medical imaging for other purposes or through surgery for other conditions. In our case, the patient presented with anemia due to obscure bleeding.

Many studies emphasize the CD117 and CD 34 expression in GISTs [8]. A method of measuring of how fast a GIST is growing is by the mitotic count visual examination of a set number of ‘high power fields’ (HPF) under the microscope to count the number of tumour cells caught in the process of mitosis or cell division. The higher the mitotic count, the faster the tumor is growing. In this reported case, GIST was diagnosed as a malignancy with moderate level of phenomorphism and mitotic activity while immunohistochemical staining for CD117, SMA, and S-100 protein was positive.

The treatment of choice is the complete resection of the tumour. The surgeon’s approach in an actual case depends on factors such as: the exact anatomical site of the GIST, the characteristics of the individual patient’s particular situation, the specific location of the tumour relative to the blood supply of the involved organ, and the patient’s condition. Patients with unresectable tumours or with metastatic disease are treated with Kit/PDGFRA tyrosine kinase inhibitors. Metastases may develop in the liver and the abdominal membranes. Patients with advanced GISTs which progress rapidly and result in organ destruction have poor prognosis.

In this reported case, the used diagnostic tools were abdominal US, upper gastrointestinal endoscopy, colonoscopy, abdominal CT, VCE and angiography. The first three investigations were unremarkable due the location of the tumour. The abdominal CT correctly imaged the location and the size of the tumour. It also excluded liver or peritoneal metastases and evaluated the extension of the primary tumour. However, CT did not establish the histological diagnosis of GIST. It is known that as most GISTs have an exophytic growth, CT imaging is more useful than endoscopy and barium studies to evaluate the extension and the size of the tumour [9]. It is also sensitive for the detection of metastatic lesions [10]. Lupescu et al [11] reported that using the CT we can only suspect the diagnosis to GISTs. They refer that it is difficult to differentiate, using only CT imaging, this type of tumours from other soft-tissue ones. Finally, Wu et al in a series of 100 small intestine GISTs refer that the sensitivity rate of abdominal CT was 91% [12].

VCE revealed an extensive venous plexus giving the possible diagnosis of angiodysplasia of small intestine in our patient. This investigation did not establish the diagnosis of tumour referring simply a benign abnormality. VCE did not help the diagnosis in our case. In 29 centres of 10 European countries, 5129 patients underwent VCE. 124 (2.4%) of them had small bowel tumours [13]. The most common indication for VCE was obscure gastrointestinal bleeding (108 patients) while the main primary small bowel tumour type was GIST (32%). These data suggested that VCE detected small-bowel tumours in a small proportion of patients underwent this examination. However, the early use of this diagnostic tool could shorten the diagnostic work-up and influenced the subsequent management of these patients [13]. Ziegler et al [14] reported that VCE identified small intestine lesions generally beyond the reach of push enteroscopy in 25% to 50% of patients and revealed additional diagnostic findings in 25% of patients compared with small bowel barium radiographic studies. The authors conclude that in some cases, the information provided by VCE in patients with obscure gastrointestinal bleeding can lead to changes in management that would improve their outcome. However, we did not use push enteroscopy and small intestine barium radiographic studies so we have not the ability to compare the results among these diagnostic tools.

In this reported case, angiography showed voluminous disorder with abnormal arterial structures and areas of stagnation of the contrast medium giving the diagnosis of a hypervascular malignant tumour without establishing the histological diagnosis of GIST. Despite there are not many studies regarding the use of VCE in diagnosis of GISTs, Fang et al [15] recently supported that VCE differentiate benign from malignant tumours, define their size, range and origin, despite the exophytic or endophytic growth of the tumours. However, they do not suggest routine use of VCE because of its risk and relative complications.

In conclusion, abdominal CT, VCE, and angiography were the diagnostic approaches we used for the GIST in our case report. It is known that the sensitivity of these investigations varied according to the location of the tumour. We found that CT was the most effective diagnostic method and we suggest routine use of this procedure in opposition to VCE and angiography which can give us some information but can also lead to changes in management that would not improve patient’s outcome.

## List of abbreviations

CT: Computed tomography
GISTs: Gastrointestinal stromal tumours
HPF: High power fields
Ht: Hematocrit
PDGFRA: Platelet-derived growth factor receptor alpha
PLT: Platelets
SMA: Smooth-muscle actin
US: Ultrasonography
VCE: Video capsule endoscopy
